# Supplementary material for: Depression and frailty in older adults: A population-based cohort study
Source: PLoS One. 2021 Mar 4;16(3):e0247766. doi: 10.1371/journal.pone.0247766 (PMC7932072; doi:10.1371/journal.pone.0247766)
Supplement: S1 Table — (DOCX) [file pone.0247766.s001.docx]

**S1 Table. Poisson multiple regression model: initial and final models by the backward regression method.**

|  | **Unadjusted IRR** |  | **Initial model** | **p-value** |  | **Final model** | | **p-value** | |
| --- | --- | --- | --- | --- | --- | --- | --- | --- | --- |
|  |  | | | | | |  | |  |
| Depression | 1.29 (1.26;1.32) | | 1.18 (0.59;2.37) | 0.643 |  | 1.21 (0.63;2.36) | | 0.581 | |
| Sex (female) |  |  | 0.72 (0.41;1.25) | 0.239 |  | 0.81 (0.46;1.44) | | 0.474 | |
| Age group: |  |  |  |  |  |  | |  | |
| 70 to 79 |  |  | 2.47 (1.32;4.62) | 0.005 |  | 2.52 (1.41;4.50) | | 0.002 | |
| 80+ |  |  | 5.24 (3.08;8.91) | 0.000 |  | 4.92 (2.91;8.34) | | 0.000 | |
| Marital Status (without partner) | |  | 1.35 (0.75;2.43) | 0.317 |  |  | |  | |
| Years of schooling (under four) | |  | 1.06 (0.59;1.92) | 0.838 |  | 1.22 (0.68;2.16) | | 0.499 | |
| Self-rated health status: |  |  |  |  |  |  | |  | |
| Regular |  |  | 1.41 (0.85;2.34) | 0.185 |  |  | |  | |
| Poor and very poor |  |  | 1.49 (0.48;4.62) | 0.485 |  |  | |  | |
| BMI: |  |  |  |  |  |  | |  | |
| Low |  |  | 0.86 (0.40;1.83) | 0.689 |  |  | |  | |
| High |  |  | 1.82 (1.05;3.14) | 0.032 |  |  | |  | |
| Number of chronic diseases: |  |  |  |  |  |  | |  | |
| 1 or 2 |  |  | 1.16 (0.49;2.72) | 0.737 |  | 1.35 (0.58;3.17) | | 0.483 | |
| 3 and more |  |  | 1.32 (0.47;3.70) | 0.591 |  | 1.81 (0.67;4.93) | | 0.240 | |

**Initial model**: adjusted by sex, age group, marital status, years of schooling, self-rated health, BMI and number of chronic diseases.

**Final model**: adjusted by sex, age group, years of schooling and number of chronic diseases.
