# Supplementary material for: Depression and frailty in older adults: A population-based cohort study
Source: PLoS One. 2021 Mar 4;16(3):e0247766. doi: 10.1371/journal.pone.0247766 (PMC7932072; doi:10.1371/journal.pone.0247766)
Supplement: S2 Table — (DOCX) [file pone.0247766.s002.docx]

**S2 Table. Poisson multiple regression model considering the variable basic activities of daily living: initial and final models, by the backward regression method.**

|  | **Unadjusted IRR** | | **Initial model** | **p-value** |  | **Final model** | **p-value** |
| --- | --- | --- | --- | --- | --- | --- | --- |
|  |  | | | | | |  |
| Depression | 1.29 (1.26;1.32) | | 1.02 (0.50;2.08) | 0.955 |  | 0.98 (0.49;1.96) | 0.959 |
| Sex (female) |  |  | 0.63 (0.36;1.12) | 0.113 |  | 0.69 (0.38;1.24) | 0.210 |
| Age group: |  |  |  |  |  |  |  |
| 70 to 79 |  |  | 2.27 (1.19;4.13) | 0.013 |  | 2.40 (1.32;4.36) | 0.004 |
| 80+ |  |  | 4.48 (2.63;7.61) | 0.000 |  | 4.42 (2.57;7.61) | 0.000 |
| Marital status (without partner) |  |  | 1.34 (0.76;2.37) | 0.306 |  |  |  |
| Years of schooling (under 4) |  |  | 1.16 (0.64;2.10) | 0.628 |  | 1.27 (0.71;2.27) | 0.407 |
| Self-rated health: |  |  |  |  |  |  |  |
| Regular |  |  | 1.18 (0.68;2.02) | 0.554 |  |  |  |
| Poor and very poor |  |  | 1.14 (0.40;3.26) | 0.809 |  |  |  |
| BMI: |  |  |  |  |  |  |  |
| Low |  |  | 0.99 (0.47;2.06) | 0.974 |  |  |  |
| High |  |  | 1.64 (0.94;2.87) | 0.084 |  |  |  |
| Number of chronic diseases: |  |  |  |  |  |  |  |
| 1 or 2 |  |  | 1.09 (0.45;2.64) | 0.85 |  | 1.20 (0.50;2.87 | 0.679 |
| 3 and more |  |  | 1.02 (0.33;3.13) | 0.968 |  | 1.27 (0.43;3.81) | 0.661 |
| Difficulty in the basic activities of daily living |  |  | 2.85 (1.51;5.35) | 0.001 |  | 3.07 (1.65;5.73) | 0.001 |

**Initial model**: adjusted by sex, age group, marital status, schooling, self-rated health, BMI, number of chronic diseases, and difficulty in the BADL.

**Final model**: adjusted by sex, age group, years of schooling, number of chronic diseases and difficulty in the BADL.
