# Supplementary material for: Depression and frailty in older adults: A population-based cohort study
Source: PLoS One. 2021 Mar 4;16(3):e0247766. doi: 10.1371/journal.pone.0247766 (PMC7932072; doi:10.1371/journal.pone.0247766)
Supplement: Attachment — Submitted filename: Response to Reviewers.docx [file pone.0247766.s003.docx]

Professor Yiqiang Zhan

Academic Editor

Plos One

28 January, 2021

Dear Professor Yiqiang Zhan,

We appreciate the opportunity to revise and resubmit our manuscript PONE-D-20-35568 (*Depression and Frailty in Older Adults: A Population-Based Cohort* *Study*). My coauthors and I hereby resubmit a revised version of our paper. We provide a point-by-point response letter to each reviewer separately.

We again thank you for your guidance and the opportunity to improve our paper.

Sincerely,

Fabiana Da Mata.

**Reviewer #1**

1) **Need to specify numbers and percentage for each variables not only percentage.**

**Reply**: Thank you for your comment. We included numbers in the tables where the information was missing (Table 3 and Table 4).

2) **The p value in the final sample should be for each variable to identify significant confounders in logistic regression.**

**Reply**: We have added the p-values to the regression tables in the Supporting Information (S1 Table and S2 Table).

3) **Alphabetical and grammatical adjustment**.

**Reply**: Thank you for the suggestion. The paper has been reviewed by a professional English proofreader, who has revised the writing of the paper.

**Reviewer #2**

1) **There is a small error on page 9, line 194, the value 1119 should be corrected to 1129 (1423 - 280 -14).**

**Reply**: Thank you for this comment. When revising the paper, we noticed that the error refers to a typo in the sample size, which should be 1,413 instead of 1,423. We have changed this in the line 273.

2) **As the only suggestion I would recommend the inclusion of a comment on the possible impact of the loss of segment of individuals aged 80 and over. The loss in this segment was proportionally higher and at the same time it is the age group with the highest incidence ratio in the univariate analysis.**

**Reply**: We thank you for this suggestion. We have added a discussion of this point in the line 531.
